# From Corsica to Britain: new outdoor records of Ocnerodrilidae (Annelida: Clitellata) in western Europe

**DOI:** 10.3897/BDJ.1.e985

**Published:** 2013-10-28

**Authors:** Emilia Rota

**Affiliations:** † University of Siena, Siena, Italy

**Keywords:** *Eukerria saltensis*, *Ocnerodrilus occidentalis*, Ocnerodrilidae, England, France, northern Italy, new records, climate changes, transport vectors

## Abstract

The ocnerodrilids *Eukerria saltensis* (Beddard, 1895) and *Ocnerodrilus occidentalis* Eisen, 1878 are reported for the first time from outdoor localities above 42° N in Europe. The present new records comprise the first ever from England (River Thames, central London) and from France (River Golo, northern Corsica) and the northernmost occurrences in Italy (Liguria and Veneto). The new latitudinal limits and the numerosity of outdoor records suggest that the current environmental and climate changes are substantially enhancing the dispersal and survival possibilities of these worms, even though different transport vectors seem to be involved for the two species.

## Introduction

The Ocnerodrilidae Beddard, 1891 are a family of semiaquatic megadriles, generally filiform and smaller than 100 by 2 mm. Chaetae are eight per segment and are seldom specialized as genital chaetae; spermathecal, female and male pores are paired and occur in that order; the male pores (mostly in XVII or XVIII) are more or less closely associated with the openings of paired, long, tubular prostates. Dorsal pores are generally absent. The digestive system has a short preintestinal region (I-XI), endowed or not with gizzards (VII-VIII) and extramural calciferous glands (IX-X), the nephridia are holoic and avesiculate, and the last pair of hearts occur in XI. The phylogenetic position of the Ocnerodrilidae in relation to the other megascolecoid families has been historically controversial, partly depending on the range of taxa considered, and partly on the authors' subjective evaluation of facts at hand (see Blakemore 2005). Michaelsen (1900) (and followers) saw the Acanthodrilidae as the most primitive within the Megascolecidae s.l. and hypothesized (Michaelsen 1921) the derivation of the Ocnerodrilidae from acanthodriline ancestors. Jamieson (1971) and Jamieson (1974) proposed an opposite view, with the ocnerodrilids at the most basal position in the megascolecoid lineage, particularly because of their morphological simplicity. Blakemore (2000) relaunched the classical view, showing that morphologically the Ocnerodrilidae are most closely related to the Acanthodrilidae with the Megascolecidae s.s. further derived. Three recent molecular investigations of earthworm phylogeny (Jamieson et al. 2002, Pop et al. 2005, James and Davidson 2012), having each analysed about 30 megascolecoid species, including up to four different ocnerodrilines (though not the family type genus *Ocnerodrilus*), agree in suggesting that the Ocnerodrilidae are the sister taxon to the Megascolecidae s.l.

**Figure 1.**
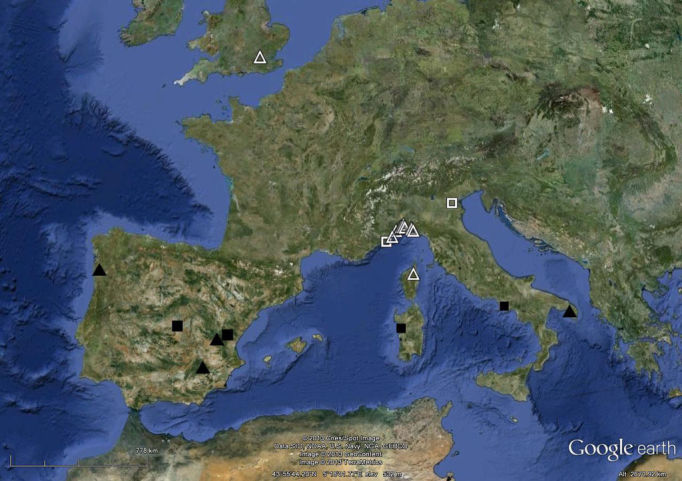
Map of outdoor records of *Eukerria saltensis* (triangles) and *Ocnerodrilus occidentalis* (squares) in Europe. White hollow symbols indicate the new localities. Courtesy Google Earth.

All of the endemic ocnerodrilid genera and species are confined to South America, sub-Saharian Africa, and India but several peregrine species are currently distributed at tropical and subtropical latitudes worldwide: *Nematogenia panamensis* (Eisen, 1900) and *Gordiodrilus elegans* Beddard, 1892 both possibly of African origin; *Eukerria saltensis* (Beddard, 1895) and *Eukerria kukenthali* (Michaelsen, 1908), native to South America, and *Ocnerodrilus occidentalis* Eisen, 1878, of uncertain Central American or Central African origin. In Europe, outdoor records of this family had so far been restricted to collections of *Eukerria saltensis* and *Ocnerodrilus occidentalis* in southern countries (Spain, Portugal and Italy; Rota 2011), although early authors documented the arrival of several distinct taxa in botanical gardens further north (e.g. 8 species now classified in 4 genera arrived in Kew’s glasshouses with plants from Africa and the Caribbean; Beddard 1891, Beddard 1892, Beddard 1893b, Beddard 1893a). Thus, the cooler parts of Europe were so far regarded as inhospitable to these worms, even to peregrine species, except for transitory occurrence in sheltered habitats (e.g. Gates 1972, Csuzdi et al. 2008). In this paper, several new outdoor finds of *Eukerria saltensis* and *Ocnerodrilus occidentalis* are reported, from up to the Thames in central London (51°30’N), i.e. well beyond the previous outdoor limit in Europe (42°N) (Fig. 1). It is here suggested that the current environmental and climate changes are responsible for a substantial increase of these worms’ dispersal and survival possibilities, which may cause an accelerated spread of them through the continent. Neither of the two species, however, seem to pose serious threats to the indigenous European earthworm fauna.

## Materials and methods

The collection in the River Thames (UK) occurred during a benthic survey carried out by Emu Ltd in 2000, covering both intertidal and subtidal areas. Intertidal cores were collected using a PVC pipe sampler (12 cm ID, 50 cm long), subtidal samples were taken by means of a small grab from a vessel (Dr Steve Jarvis, in litteris). The worms were passed to me, partly in alcohol and partly sectioned on slides, by Dr Tarmo Timm (Estonia). The specimens from River Golo (Corsica) were extracted alive in Siena from moist grey sand sampled on the river bank and carried to Siena in PVC vials by Prof. Folco Giusti. All the material from Liguria, Italy, was collected by Dr Marco Bodon and colleagues during regional water quality monitoring programs using standard macroinvertebrate methods for rivers (handnet and Surber net sampling). The many occurrences in Liguria cover a variety of situations: perennial and intermittent water courses, stream beds with or without vegetation, gravelly or sandy-muddy bottoms, with slopes 0.2-3.5%, at a distance of up to 4.2 km from the river mouth (altitude 1-148 m a.s.l.), in the proximity or not of agricultural, domestic and industrial waste and sewage inputs. At the time of collection, the wet river bed at the various stations ranged between 2-40 m in width, the mean depth 10-35 cm, pH 7.9-8.4, conductivity 187-390 µS/cm, and IBE values ranged overall between 5 and 9-10. Adult specimens were sampled from April through November. The collection in Veneto, Italy, was part of an unpublished 1981-1983 survey of the earthworm fauna populating the center of the Veneto region. The finding occurred in an area of warm saline springs surrounded to the west by the Euganean Hills and to the east by a network of waterways that flow downwards to the Adriatic sea. The worms were collected manually by digging at a distance of 0.5-5.0 m from a thermal ditch.

## Taxon treatments

### Eukerria saltensis

(Beddard, 1895)

#### Materials

- **Occurrence:** recordedBy: S. Jarvis; individualCount: 3; **Location:** waterBody: River Thames; country: England; verbatimLocality: Central London, banks between Lambeth and Vauxhall Bridges; verbatimCoordinates: 51°29'33"N, 0°7'20"W; **Event:** eventDate: August 2000
- **Occurrence:** recordedBy: F. Giusti; individualCount: 10; **Location:** waterBody: River Golo; island: Corsica; country: France; verbatimLocality: below Hotel Accendi Pipa, 5 km west of Barchetta; verbatimElevation: 125 m a.s.l; verbatimCoordinates: 42°30'17"N, 9°19'7"E; **Event:** eventDate: 21-Jun-2006
- **Occurrence:** recordedBy: M. Bodon et al.; individualCount: 1; lifeStage: adult; **Location:** waterBody: River Centa; country: Italy; stateProvince: Savona; municipality: Albenga; verbatimElevation: 4 m a.s.l.; verbatimCoordinates: 44°2'53"N, 8°12'38"E; **Event:** eventDate: 8-Apr-2010
- **Occurrence:** recordedBy: M. Costa & al.; individualCount: 16; **Location:** waterBody: Torrente Quiliano; country: Italy; stateProvince: Savona; municipality: Valleggia; verbatimElevation: 5 m a.s.l.; verbatimCoordinates: 44°16'58"N, 8°26'27"E; **Event:** eventDate: 7-Apr-2011
- **Occurrence:** recordedBy: M. Costa & al.; individualCount: 17 (3 subadults); **Location:** waterBody: Torrente Quiliano; country: Italy; stateProvince: Savona; municipality: Valleggia; verbatimElevation: 5 m a.s.l.; verbatimCoordinates: 44°16'58"N, 8°26'27"E; **Event:** eventDate: 11-May-2011
- **Occurrence:** recordedBy: M. Bodon; individualCount: 1; lifeStage: juvenile; **Location:** waterBody: Torrente Lerone; country: Italy; stateProvince: Genova; municipality: Cogoleto; verbatimLocality: near the entrance to former Stoppani chemical company; verbatimElevation: 10 m a.s.l.; verbatimCoordinates: 44°23'31"N, 8°39'54"E; **Event:** eventDate: 17-Oct-2007
- **Occurrence:** recordedBy: S. Gaiter; individualCount: 1; lifeStage: adult; **Location:** waterBody: Torrente Cerusa; country: Italy; stateProvince: Genova; municipality: Voltri; verbatimElevation: 17 m a.s.l.; verbatimCoordinates: 44°25'44"N, 8°44'33"E; **Event:** eventDate: 5-May-1992
- **Occurrence:** recordedBy: D. Rocca & S. Amabene; individualCount: 6; **Location:** waterBody: Torrente Leira; country: Italy; stateProvince: Genova; municipality: Voltri; verbatimElevation: 4 m a.s.l.; verbatimCoordinates: 44°25'55"N, 8°44'58"E; **Event:** eventDate: 5-Apr-2007
- **Occurrence:** recordedBy: D. Rocca; individualCount: 10; **Location:** waterBody: Torrente Leira; country: Italy; stateProvince: Genova; municipality: Voltri; verbatimElevation: 4 m a.s.l.; verbatimCoordinates: 44°25'55"N, 8°44'58"E; **Event:** eventDate: 11-Oct-2007
- **Occurrence:** recordedBy: S. Arioni & M. Iorio; individualCount: 1; lifeStage: adult; **Location:** waterBody: Torrente Chiaravagna; country: Italy; stateProvince: Genova; municipality: Sestri Ponente; verbatimElevation: 8 m a.s.l.; verbatimCoordinates: 44°25'37"N, 8°51'14"E; **Event:** eventDate: 7-Jun-2009
- **Occurrence:** recordedBy: D. Rocca & S. Amabene; individualCount: 10; **Location:** waterBody: Rio d’Iso, Campora; country: Italy; stateProvince: Genova; municipality: Campora; verbatimElevation: 148 m a.s.l.; verbatimCoordinates: 44°31'9"N, 8°52'26"E; **Event:** eventDate: 14-May-2007
- **Occurrence:** recordedBy: D. Rocca & S. Amabene; individualCount: 2; **Location:** waterBody: Rio d’Iso, Campora; country: Italy; stateProvince: Genova; municipality: Campora; verbatimElevation: 148 m a.s.l.; verbatimCoordinates: 44°31'9"N, 8°52'26"E; **Event:** eventDate: 13-Sep-2007
- **Occurrence:** recordedBy: S. Arioni; individualCount: 10; **Location:** waterBody: Torrente Bisagno; country: Italy; stateProvince: Genova; municipality: Ponte S. Agata; verbatimElevation: 6 m a.s.l.; verbatimCoordinates: 44°24'29"N, 8°57'2"E; **Event:** eventDate: 2-Jun-2008
- **Occurrence:** recordedBy: M. Bodon; individualCount: 1; lifeStage: adult; **Location:** waterBody: Torrente Lavagna; country: Italy; stateProvince: Genova; municipality: S. Pietro di Sturla; verbatimElevation: 22 m a.s.l.; verbatimCoordinates: 44°21'45"N, 9°19'28"E; **Event:** eventDate: 3-May-1993
- **Occurrence:** recordedBy: D. Rocca & R. Farinelli; individualCount: 2; lifeStage: juvenile; **Location:** waterBody: River Entella; country: Italy; stateProvince: Genova; municipality: Lavagna; verbatimLocality: Ponte Maddalena; verbatimElevation: 4 m a.s.l.; verbatimCoordinates: 44°19'4"N, 9°20'38"E; **Event:** eventDate: 26-Apr-2007
- **Occurrence:** recordedBy: D. Rocca & S. Amabene; individualCount: 1; **Location:** waterBody: River Entella; country: Italy; stateProvince: Genova; municipality: Lavagna; verbatimLocality: Ponte Maddalena; verbatimElevation: 4 m a.s.l.; verbatimCoordinates: 44°19'4"N, 9°20'38"E; **Event:** eventDate: 4-Sep-2007
- **Occurrence:** recordedBy: D. Rocca & S. Amabene; individualCount: 10; **Location:** waterBody: Torrente Petronio; country: Italy; stateProvince: Genova; verbatimLocality: upstream of Riva Trigoso; verbatimElevation: 10 m a.s.l.; verbatimCoordinates: 44°15'47"N, 9°25'29"E; **Event:** eventDate: 10-Apr-2007
- **Occurrence:** recordedBy: D. Rocca & S. Amabene; individualCount: 2; **Location:** waterBody: Torrente Petronio; country: Italy; stateProvince: Genova; verbatimLocality: upstream of Riva Trigoso; verbatimElevation: 10 m a.s.l.; verbatimCoordinates: 44°15'47"N, 9°25'29"E; **Event:** eventDate: 2-Nov-2007
- **Occurrence:** recordedBy: D’Arena & al.; individualCount: 4; **Location:** waterBody: Torrente Petronio; country: Italy; stateProvince: Genova; verbatimLocality: upstream of Riva Trigoso; verbatimElevation: 10 m a.s.l.; verbatimCoordinates: 44°15'47"N, 9°25'29"E; **Event:** eventDate: 11-May-2011
- **Occurrence:** recordedBy: M. Costa & al.; individualCount: 7; **Location:** waterBody: Torrente Petronio; country: Italy; stateProvince: Genova; verbatimLocality: upstream of Riva Trigoso; verbatimElevation: 10 m a.s.l.; verbatimCoordinates: 44°15'47"N, 9°25'29"E; **Event:** eventDate: 19-Jul-2011
- **Occurrence:** recordedBy: M. Costa & al.; individualCount: 3; **Location:** waterBody: Torrente Petronio; country: Italy; stateProvince: Genova; verbatimLocality: upstream of Riva Trigoso; verbatimElevation: 10 m a.s.l.; verbatimCoordinates: 44°15'47"N, 9°25'29"E; **Event:** eventDate: 30-Aug-2011

#### Diagnosis

**Figure 2.**
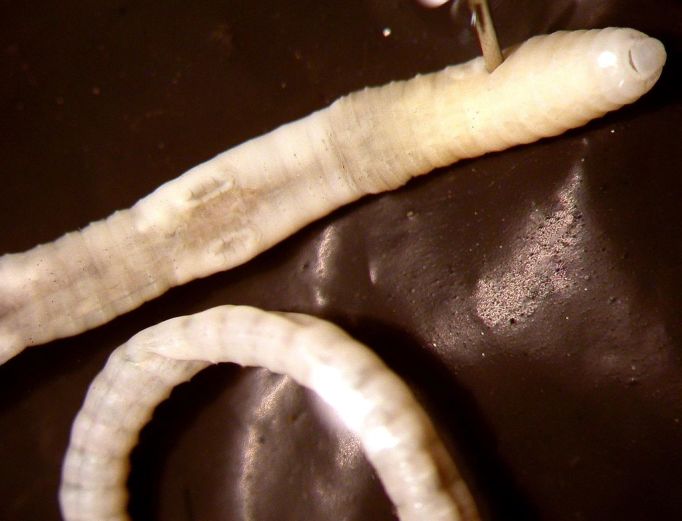
*Eukerria saltensis*, anterior part of the body (ventral view) and tail’s coil, showing the two typical longitudinal grooves connecting the prostate (in XVII, XIX) and male pores (XVIII).

Body size 30-70 x 1-1.8 mm. Segment number 70-130. Easily identified by the annular clitellum covering XIII-XX and marked ventrally by two longitudinal grooves connecting the prostate pores (in XVII, XIX) and male pores (in XVIII) of each side (Fig. 2). A gizzard occurs in VII, and the calciferous glands appear as paired sausage-shaped diverticula originating posteriorly in IX. Paired spermathecae in VIII and IX, opening in the anterior intersegment below chaetal line *c*. The thin tubular prostates, when fully distended, occupy 3-7 segments.

### Ocnerodrilus occidentalis

Eisen, 1878

#### Materials

- **Occurrence:** recordedBy: L. Braida & T. Braida; individualCount: 2; lifeStage: adults; **Location:** waterBody: Torrente Argentina; country: Italy; stateProvince: Liguria; county: Imperia; municipality: Taggia; verbatimLocality: bed of Torrente Argentina; verbatimElevation: 26 m a.s.l.; verbatimDepth: 40 cm; verbatimCoordinates: 43°50'56"N, 7°51'34"E; **Event:** eventDate: 08-Dec-2006; eventRemarks: along with Microscolex phosphoreus (1 adult)
- **Occurrence:** recordedBy: L. Braida & T. Braida; individualCount: 1; lifeStage: juvenile; **Location:** waterBody: Torrente Argentina; country: Italy; stateProvince: Liguria; county: Imperia; municipality: Taggia; verbatimLocality: bed of Torrente Argentina; verbatimElevation: 26 m a.s.l.; verbatimDepth: 40 cm; verbatimCoordinates: 43°50'56"N, 7°51'34"E; **Event:** eventDate: 08-Dec-2006; eventRemarks: along with Microscolex phosphoreus (1 adult)
- **Occurrence:** recordedBy: P. Omodeo & P. Negrisolo; individualCount: 1; **Location:** waterBody: Battaglia Terme; country: Italy; stateProvince: Padova; municipality: Veneto; verbatimLocality: edge of thermal water ditch bordering grassfield outside Villa Selvatico, between the railway and former “Pietro d’Abano” spa; verbatimCoordinates: 45°17'10"N, 11°46'34"E; **Event:** eventDate: September 1982; eventRemarks: along with Dichogaster modiglianii (1 adult) and Microscolex phosphoreus (5 adults)

#### Diagnosis

**Figure 3.**
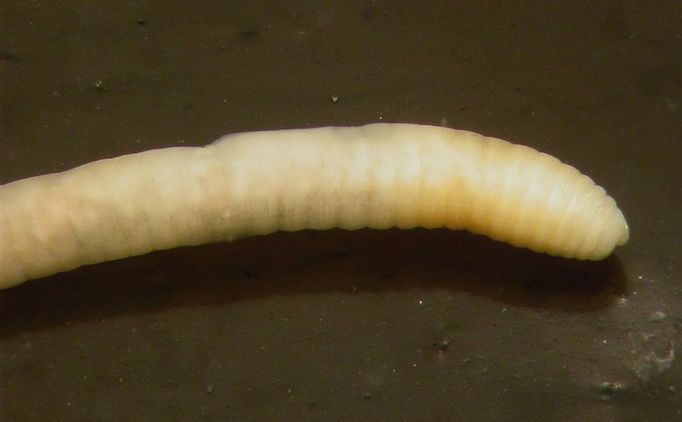
*Ocnerodrilus occidentalis*, anterior part of the body (ventrolateral view), showing the two small prominent male porophores in XVII.

Body size 30-40 x 1-1.4 mm. Segment number 60-80. Easily identified by the annular clitellum covering XIII-XIX or XIII-XX marked ventrally by small paired male porophores on XVII (Fig. 3); the latter generally connected by a ventral transverse groove. No gizzard. Calciferous glands as large paired fan-shaped diverticula originating posteriorly in IX. Spermathecae absent. Prostates one pair, much elongated (can reach XXIV), opening close to male pores.

## Discussion

### Habitat, possible sources and dispersal of *Eukerria saltensis*

In Europe *Eukerria saltensis* had so far been collected outdoors in Spain (Diaz-Cosín et al. 1980b, Mariño et al. 1986, Martinez-Lopez et al. 1990, Onteniente and Babío 2009), Portugal (Trigo et al. 1988) and Italy (Ferreri 1996). The present new records comprise the first ever from England (River Thames, central London) and France (Corsica, River Golo) and the northernmost locations in Italy, scattered all along the coast of Liguria (Fig. 1).

A survey of the literature suggests the ability of this species to colonize diverse habitats through multiple pathways. Records in non-native regions range from well-preserved natural water bodies to artificial wet biotopes (man-made water catchments, flooded or irrigated areas, zoological gardens) or degraded sites along streams or rivers (e.g. Plisko 2010). This species is usually found between the roots of riparial or aquatic plants but, as the new records confirm, it can tolerate highly degraded situations. It was never intercepted during 32 years of inspection for quarantineable plant pests at US ports of entry (see Gates 1982). It evidently requires high moisture and sufficient aeration for survival during transportation, but also careful handling because of its body’s fragility (Blakemore et al. 2006). About 20 km upstream of Lambeth Bridge in London, the Thames flows just outside Kew Gardens and this or some other artificial biotopes could be the source for the Lambeth *Eukerria* population. Friend (1916) described his *Kerria rubra* - possibly a *species dubia* (Jamieson 1970) and in any case not a synonym of *Eukerria saltensis* (because lacking a gizzard) – on specimens found in the tropical water lily tank at the Oxford Botanical Garden. Over the last 20–30 years, the Thames has shown an overall increase in water temperature of 1–2°C, with the highest values seen from 1990 to present (Hammond and Pryce 2007). This certainly facilitates outdoor survival and adaptability of organisms native to warmer climates. For instance, the Asiatic tubificoid oligochaete *Branchiura sowerbyi*, first found outdoors in the vicinity of a power station near Reading (Mann 1958), has taken up residence within the London city boundaries and represents in places a major component of the Thames macrozoobenthos (Attrill 1998).

River traffic and port activities have made the Thames catchment one of the most highly invaded freshwater systems in the world, particularly in the London area (Jackson and Grey 2012). Although much more isolated, Corsican inland waters have been also exposed to the introduction of nonindigenous species. Suffice it to say that the *Eukerria* site is one of the many recording stations of the New Zealand gastropod *Potamopyrgus antipodarum* in the island (Favilli et al. 1998, Zettler and Richard 2004). The many occurrences of *Eukerria saltensis* in Liguria probably result from concurrent causes: intense port activities; floriculture and horticultural production and trade (practised all along the coast and in the immediate hinterland with terraced glasshouses); exotic gardening; freshwater angling. Several Liguria watercourses, including T. Lavagna and R. Entella mentioned above for *Eukerria saltensis*, harbour today populations of *Branchiura sowerbyi*, while *Potamopyrgus antipodarum* can now be collected virtually everywhere in Liguria (M. Bodon in litt.).

Floods and roads (i.e. human activities involving downhill and uphill transport) must play an important role in accelerating the local dispersal of this species. Both the River Golo and the majority of watercourses in Liguria are characterised by a torrential mode with short and violent floods. The former record from Italy (Ferreri 1996; erroneously stated as the first European record) occurred on the muddy banks of Vora Colucci (Nardò, Lecce Province, Apulia), a karstic sinkhole draining a man-made network of natural and artificial channels (called the Asso system) affected annually by flooding. Taiwan, a mountainous island country often confronted with floods, but also with intense forestry and watershed management activities (Lu et al. 2001), offers perhaps the most spectacular example of human-mediated dispersal of *Eukerria saltensis*: the species can be found there up to altitudes of 1770 m a.s.l. and especially along the sides of forest roads (Shen et al. 2008).

### Habitat, possible sources and dispersal of *Ocnerodrilus occidentalis*

The only outdoor records of *Ocnerodrilus occidentalis* in Europe were from Italy (Gates 1973, Omodeo 1984) and Spain (Diaz-Cosín et al. 1980a, Jesús et al. 2002), plus the Canary Islands (Talavera 1990). The Italian records included nearly 60 specimens collected in 1925 in Naples (Posillipo and Agnano, mud) by the then 22 year old G. Evelyn Hutchinson (Gates 1973), and one adult from Sardinia (Omodeo 1984) published without locality. Details of the latter in Omodeo’s notes are as follows: Sardinia, Oristano Province, near Bosa, on the banks of River Temo (40°17'43"N, 8°31'51"E, P. Omodeo & G. Valbusa coll., 6-Oct-1980), in black, smelly, anoxic submerged soil at the margins of orchards and olive groves, tamerix and willows. The continental Spanish records were from the bottom of irrigation channels (Diaz-Cosín et al. 1980a, Jesús et al. 2002) and the Canarian ones involved a variety of cultivated fields (tropical plantations, alfalfa, tomato plots) as well as gardens, pastures and xerophilous scrubland (Talavera 1990).

The present new records are the first from northern Italy (Veneto and Liguria). The recording site in Liguria was near an area of floral trading companies (e.g. MFI Italia Esportazione Fiori). Gates (1973) and Gates (1979) listed U.S. quarantine interceptions of *Ocnerodrilus occidentalis* in soil accompanying a variety of potted plants: ferns, cactus, *Bougainvillea*, *Philodendrum*, *Citrus*, *Pedilanthus*, *Musa*, *Tropaeolum peltophorum*, oleanders, orchids, iris, lemon grass, *Polianthus*, as well as mango seeds and potato tubers. None of these plants is aquatic. Csuzdi et al. (2008) found it abundant in a tropical plant nursery and at greenhouses in Hungary. Other records presumably from European greenhouses include: specimens intercepted by Gates (1973) and Gates (1979) in potted plants from Italy, Greece and Madeira and with potato tubers from Germany. An interception from Denmark referred by the same author to an unspecified (unidentifiable) 1964 publication is probably a lapsus.

### Conclusions

Increasing international trade and human-induced environmental changes are multiplying the chances, modes and pathways of dispersal of exotic earthworm species in Europe. The case of an epigeic tropical megascolecid s.l., *Dichogaster bolaui* (Michaelsen, 1891), which, via house plants gardening, has now become an habitué of indoor plumbing systems in the cooler parts of the continent, has been reviewed by Rota and Schmidt (2006) and Csuzdi et al. (2008). Both the ocnerodrilid species dealt with in this paper are commonly found in rice farms and have been suggested to have initially expanded their distribution through rice cultivation (Rota 2011). *Ocnerodrilus occidentalis* is an amphibious species, tolerant of hypoxia and low moisture and, as documented by border interceptions (Gates 1982), can be spread like *Dichogaster bolaui* as contaminant of virtually any kind of ornamentals and horticultural products. *Eukerriasaltensis* is seldom terrestrial and much more sensistive to desiccation. It survives periods of soil dryness primarily in the cocoon stage (Stevens 2000), so it may occasionally travel the same routes as *Ocnerodrilus occidentalis* without our notice. However, its main human-mediated vectors are likely to be crops grown in wet or flooded fields and ornamentals for watergardens and aquaria. Water-ways such as rivers and canals, then, would allow swift dispersal rates and act as links between different habitats, all the more so if they are frequently flooded. It may not be by chance that the new *Eukerria* records are all located in areas subject to flooding. Climate change is affecting aquatic systems by warming water temperatures, altering stream flow patterns, and increasing storm events. Thus, as suggested by Rahel and Olden (2008), climate change is expected to further influence the likelihood of warm-water species becoming established by eliminating winter hypoxia that currently prevents survival and facilitate their spread during floods.

## Supplementary Material

XML Treatment for Eukerria saltensis

XML Treatment for Ocnerodrilus occidentalis
